# Cellular Mechanisms of Zinc Dysregulation: A Perspective on Zinc Homeostasis as an Etiological Factor in the Development and Progression of Breast Cancer

**DOI:** 10.3390/nu4080875

**Published:** 2012-07-30

**Authors:** Samina Alam, Shannon L. Kelleher

**Affiliations:** 1 Department of Nutritional Sciences, The Pennsylvania State University, University Park, PA 16802, USA; Email: sra116@psu.edu; 2 Department of Surgery, the Pennsylvania State University College of Medicine, Hershey, PA 17033, USA; 3 Cell and Molecular Physiology, the Pennsylvania State University College of Medicine, Hershey, PA 17033, USA

**Keywords:** breast cancer, zinc homeostasis, oxidative stress, DNA damage/repair, transcription, zinc transport, cell cycle, proliferation, cell signaling, apoptosis

## Abstract

Worldwide, breast cancer is the most commonly diagnosed cancer among women and is the leading cause of female cancer deaths. Zinc (Zn) functions as an antioxidant and plays a role in maintaining genomic stability. Zn deficiency results in oxidative DNA damage and increased cancer risk. Studies suggest an inverse association between dietary and plasma Zn levels and the risk for developing breast cancer. In contrast, breast tumor biopsies display significantly higher Zn levels compared with normal tissue. Zn accumulation in tumor tissue also correlates with increased levels of Zn importing proteins. Further, aberrant expression of Zn transporters in tumors correlates with malignancy, suggesting that altered metal homeostasis in the breast could contribute to malignant transformation and the severity of cancer. However, studies have yet to link dysregulated Zn transport and abnormal Zn-dependent functions in breast cancer development. Herein, we summarize studies that address the multi-modal role of Zn dyshomeostasis in breast cancer with respect to the role of Zn in modulating oxidative stress, DNA damage response/repair pathways and cell proliferation/apoptosis, and the relationship to aberrant regulation of Zn transporters. We also compare Zn dysregulation in breast tissue to that of prostate, pancreatic and ovarian cancer where possible.

## 1. Introduction

**Figure 1:**
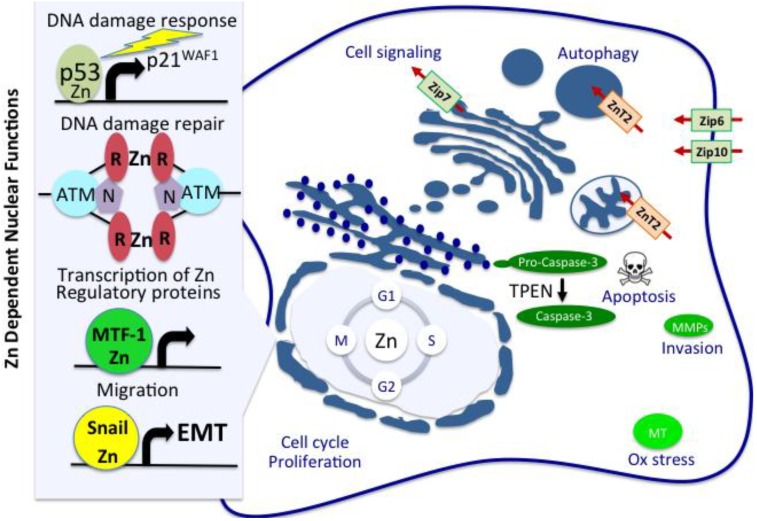
Homeostatic role of Zn in cellular functions which potentially link to initiation and progression of breast cancer. Zn is a biologically essential metal and is a cofactor for more than 300 enzymes and proteins involved in the defense against oxidative stress, including metallothionein, proteins controlling responses to DNA damage and repair (p53; ATM/MRN), intracellular signaling enzymes, more than 2000 transcription factors that require Zn for their structural stability and binding to DNA, also known as Zn-finger proteins, including MTF-1, which controls transcription of Zn regulatory proteins such as metallothionein (MT), and matrix metalloproteinases (MMPs), a family of Zn-dependent endopeptidases which regulate tissue remodeling. Zn also functions as an intracellular second messenger, cytosolic levels of which are tightly controlled by coordinate regulation of ZnTs and ZIPs. In addition, Zn is critical for cell proliferation, cell cycle regulation, differentiation and apoptosis. Cumulatively, the antioxidant and anti-carcinogenesis mechanisms associated with Zn homeostasis appear to play an inhibitory role on neoplastic cell growth.

Worldwide, breast cancer is the most commonly diagnosed cancer among women and is the leading cause of female cancer deaths [1]. In 2008, women diagnosed with breast cancer accounted for 23%, of total cancer cases, and 14% of total cancer deaths resulted from this cancer [1]. Numerous studies document both molecular and genetic factors as initiators and promoters of breast tissue oncogenesis [2]. Among these categories, deregulated mechanisms contributing to increased oxidative stress and consequent genomic instability play an important role in the development of a variety of human diseases, including breast cancer [3,4]. Inadequate nutrition translates to dietary deficiencies of key micronutrients that could manifest as increased cellular stress and associated DNA damage [5,6,7]. In this regard, Zn is an essential trace element and its functions as an antioxidant and its role in the maintenance of genomic stability have been widely reported [8]. Epidemiological studies have established a link between dietary Zn deficiency and an increased risk of developing cancer [9]. Zn deficiency is a global public health problem, leaving ~2 billion people at risk for deficiency of this trace metal [10]. In the US, almost 10% of the population does not absorb an adequate amount of Zn from the diet [10]. The global effect of Zn deficiency and its link to cancer can certainly be appreciated considering that this biologically essential metal is a component of approximately 3000 proteins (almost 10% of total proteins) [11], of which more than 300 enzymes require Zn as a cofactor [12]. Subsets of these proteins are involved in the defense against oxidative stress, including metallothionein [13], and Cu/Zn superoxide dismutase (SOD) [14], proteins controlling responses to DNA damage and repair [15,16,17,18], intracellular signaling enzymes [19,20], more than 2000 transcription factors (Zn-finger proteins) that require Zn for their structural stability and binding to DNA [21,22], and matrix metalloproteinases (MMPs), a family of Zn-dependent endopeptidases that regulate tissue remodeling [23]. Zn also functions as an intracellular second messenger [24] and is critical for cell proliferation, cell cycle regulation, differentiation and apoptosis [25,26,27,28,29]. Cumulatively, the antioxidant and anti-carcinogenesis mechanisms associated with Zn homeostasis appear to play an inhibitory role on neoplastic cell growth. In this review, we explore each of these functional categories with regard to our current understanding of the consequences resulting from Zn dysregulation in breast tissues and the development of breast cancer (Figure 1).

## 2. Comparative Plasma and Tumor Zinc Levels and Breast Cancer Risk

The role of Zn as an antioxidant has attracted considerable attention, particularly with regard to cellular controls that regulate Zn dyshomeostasis in cancer [26]. Although serum Zn levels are considered a poor indicator of Zn status, serum Zn levels are generally low in patients with multiple cancers [30], including patients with squamous esophageal cancer [31], malignant prostate cancer [32], ovarian cancer [33], and cancers of the gallbladder [34], lung [35], colon, head and neck [36], and bronchus [35]. Multiple studies also show a relationship between low plasma Zn levels and the risk for developing breast cancer [37,38,39], prompting the suggestion that plasma Zn levels can be used as a prognostic as well as a therapeutic marker for breast cancer [40,41,42]. Other studies also considered analysis of Zn levels in erythrocytes to be a more accurate indicator [43], since significantly lower Zn levels in erythrocytes in women with breast cancer compared with normal women could not be attributed to dietary Zn insufficiency [44].

Zn dyshomeostasis displays an added layer of complexity when studies are considered that address the differential regulation of serum Zn levels of cancer patients compared to its levels in the corresponding tumor tissues. Breast biopsies from cancer patients have significantly higher Zn levels compared with normal breast tissue [45,46,47,48]. Epidemiological studies have established a relationship between high breast tissue Zn levels and development of breast cancer [49]. Zn accumulation in tumor tissues is correlated with increased expression of cellular Zn importing proteins compared with normal tissues, suggesting that tumor cells selectively increase Zn uptake using common mechanisms [26]. Likewise, expression levels of Zn transporters in tumors also correlate with their malignancy, suggesting that altered Zn homeostasis could contribute to the severity of cancer [50,51,52]. Together, this supports the idea that abnormal Zn metabolism is a common link in cancer development [30]. Elevated tumor Zn levels are also characteristic of patients displaying other cancer types [30,31,53,54] supporting the idea that abnormal Zn metabolism is a common link in cancer development. Despite multiple studies that implicate altered Zn metabolism in the development of breast cancer [47,48,55,56,57], concrete evidence linking Zn dyshomeostasis as an etiological factor has yet to be revealed. In this review we discuss the current knowledge of the functional role of cellular Zn and the studies that link defects in Zn homeostasis, with alterations in mechanisms that potentially contribute to initiation and progression of breast cancer.

## 3. Zinc and Oxidative Stress

Studies in humans [9,58], animal models [59,60], and cultured cells [61] provide support for the link between Zn deficiency and its regulation of oxidative stress. Zn is an essential cofactor for the activity of a number of proteins that play a role in combatting oxidative stress, such as metallothioneins [13] and Cu/Zn SOD [62]. Zn is also a cofactor for proteins involved in mediating DNA damage response and repair, such as the p53 tumor suppressor protein [13]. Therefore, suboptimal Zn intake may promote the generation of oxidative stress, and single and double DNA strand breaks similar to radiation induced DNA damage [63,64]. Compromised DNA integrity, impaired functioning of DNA repair enzymes and loss of DNA surveillance mechanisms are associated with increased risk of cancer initiation and progression [18,25,28,65,66].

## 4. Zinc-Metallothionein Regulation of Oxidative Stress and Chemoresistance in Breast Cancer

Metallothioneins are stress-inducible proteins with antioxidant properties that protect cells against the generation of free radicals and reactive oxygen species [13]. Metallothioneins are cysteine-rich, low molecular weight proteins that have a specific and high binding affinity for biologically essential metals such as Zn and Cu [13]. Metallothioneins buffer cytosolic Zn and maintain negligible amounts of “free” cytosolic Zn [67]. Tight control of cytosolic Zn buffering is necessary for maintaining the redox status of the cells, as both increased and decreased Zn levels induce oxidative stress [68]. Sequestration of Zn via binding to redox-sensitive cysteine moieties allows metallothioneins to act as a Zn-donor or a Zn-acceptor (apo-metallothionein), which in turn allows for reversible transfer of Zn ions to many cellular proteins, such as DNA and RNA polymerases and transcription factors [69]. In this way, apo-metallothionein is known to inactivate the DNA binding capacity of Zn finger transcription factors in vitro [69,70], whereas the Zn-bound metallothioneins restore their activity [70]. Additionally, because of high cysteine content, metallothioneins defend against oxidative stress by binding to free radicals acting as potent scavenger of reactive oxygen species (ROS), such as hydrogen peroxide (H_2_O_2_), superoxide (O_2_^−^), nitric oxide (NO) and hydroxyl (OH) radicals [71]. Metallothionein-mediated scavenging of free radicals protects macromolecules such as DNA, proteins and lipids against oxidative damage from these highly reactive compounds [72,73].

Metallothionein expression is deregulated in breast cancer. High metallothionein expression correlates with chemoresistance and poor prognosis [74,75]. Metallothioneins participate in carcinogenesis through at least two mechanisms that promote the development of chemo- or radioresistant tumor cells. First, elevated metallothionein levels in cancer cells protect against free-radical damage, thereby inhibiting apoptosis and promoting cell proliferation, and supporting uncontrolled growth [76]. Secondly, metallothionein interaction with Zn ions is involved in regulating multiple transcription factors, contributing towards carcinogenesis [13,76,77]. Increased metallothionein expression is positively associated with histological grade in invasive breast ductal carcinoma [78,79], and resistance to radiation [80] as well as chemotherapeutics [81]. Invasive ductal breast carcinomas overexpress metallothionein, whereas their lobular counterparts express metallothionein weakly [82,83]. While mechanisms responsible for metallothionein deregulation in breast cancer cells are still under investigation, it has been proposed that the de-differentiated status of cancer cells could play a role in the induction of this protein [84]. Therefore, metallothionein expression could be a useful marker of less differentiated, more aggressive breast cancer phenotype [83].

## 5. Zinc Regulation of p53 Activation, ATM/MRN Mediated DNA Damage Response and Repair

Zn is required by multiple proteins in DNA replicative machinery including DNA and RNA polymerases, and transcription factors including p53 [85]. Normal cells respond to DNA damage via induction of p53 protein levels, which transcriptionally regulates multiple functions related to DNA repair, cell cycle checkpoint regulation and induction of apoptosis [86,87]. In response to DNA damage signals, p53 either mediates G1 phase cell cycle arrest by inducing transcription of the cyclin-dependent kinase inhibitor p21^WAF1^ thereby allowing time for DNA repair, or activating cell death by triggering apoptosis [88]. Zn deficiency increases oxidative DNA damage [27] and induces chromosome breaks [59,89]. Additionally, Zn deficiency increases p53 expression in response to DNA damage, but impairs the ability of p53 to bind DNA [66]. The p53 protein DNA binding domain is stabilized by a Zn ion, which is necessary for maintaining a functionally active conformation [90]. Under Zn deficient conditions, apo-metallothionein can chelate Zn from p53 and disrupt architecture of the DNA binding domain, allowing the protein to adopt a conformation identical to mutant forms of p53 [91,92] and inactivating the protein [89]. Persistent apo-metallothionein overexpression in tumor cells may promote increased proliferation and survival through promotion of cellular conditions that are essentially devoid of wild-type p53 functions [93]. It is of note that p53 mutations in breast cancer [87], often correlate with high grade and triple negative cancers [94,95].

Transcriptional activation of p53 is regulated via phosphorylation of its Ser^15^ residue, mediated by the upstream ataxia-telangiectasia mutated (ATM) protein [96]. The ATM protein itself is activated in response to DNA double-strand breaks via autophosphorylation of Ser^1981^ [97]. Interestingly, ATM is also activated in response to conditions of oxidative stress where oxidation creates a disulfide-cross-linked ATM dimer [96]. Activation of ATM is further dependent upon upstream signaling of the Mre11 (Mre11/Rad50/Nbs1) (MRN) protein complex, which is the primary sensor of DNA double-strand breaks [98]. The Mre11 complex recruits activated ATM and binds to the free ends of the damaged DNA. This bridges the broken ends of the DNA double-strand break to the CXXC sequence in the middle of Rad50, in a Zn-dependent manner [98]. These studies highlight the role of Zn in activating enzymes that regulate DNA damage response and repair. Thus Zn deficiency consequently impair DNA damage response mechanisms, facilitate the loss of DNA integrity and increase cancer risk [28].

A well known tumor suppressor BRCA1, a product of the familial breast cancer susceptibility gene, also plays a role in maintaining chromosomal stability [99]. In response to ionizing radiation, BRCA1 is phosphorylated by ATM and CHK2, followed by its recruitment to Mre11 (Rad50/Mre11/Nbs1) containing protein complexes where BRCA1 regulates chromatin remodeling, transcription regulatory factors and proteins which control cell cycle [100]. The BRCA1 protein contains an amino terminal RING finger domain containing several cysteine residues which form two potential Zn binding motifs [101]. However, Zn-dependent BRCA1 DNA binding has yet to be demonstrated. Therefore, mechanisms responsible for physically recruiting BRCA1 to regulatory sites of its transcriptional targets was a mystery until the discovery of ZBRK1, a transcription factor with multiple Zn-binding motifs, which interacts with BRCA1, and is capable of sequence specific DNA binding [100]. Recruitment of this protein complex to relevant promoters containing ZBRK1 binding motifs, such as GADD45, controls downstream activities such as growth arrest and DNA damage response/repair [100].

## 6. Zinc Regulation of Cell Proliferation, Signaling and Apoptosis

Cell cycle: Zn is an essential cofactor for cell proliferation, differentiation and apoptosis [29]. During normal cell cycle progression, Zn is requisite for G1/S transition and DNA synthesis [102,103,104,105]. Zn limitation causes DNA synthesis and growth arrest indicating that Zn is also required for S phase, thereby potentiating a growth period enriched in both cellular transcription and translation [106]. Since Zn is a structural element in a number of proteins that regulate multiple S phase functions including transcription [107], DNA synthesis [108], aminoacyl-tRNA synthesis [109], and ribosomal functions [110], Zn deficiency is also associated with S phase slow-down. Lastly, Zn is also required for the subsequent G2/M transition phase [105]. Under normal conditions, the Cdc25C is a Zn-binding metalloprotein that dephosphorylates and activates the Cyclin B/cdk1 complex which then regulates entry and progression through mitosis [111]. However, Zn chelation with the cell permeable Zn chelator, TPEN (*N*,*N*,*N*′-tetrakis(2-pyridylmethylbrethylenediamine), inhibits Cdc25C-cdk1 activity and blocks the G2/M transition phase [112]. These studies highlight the role of fluctuations in intracellular Zn levels in modulation of multiple proteins that regulate the cell cycle [113], and may thus be deregulated in breast cancer.

Cell signaling: The effect of Zn deficiency/chelation and Zn supplementation has also been investigated on mitogenic signaling pathways in response to growth factor activation [29]. Zn regulation of protein phosphatases is an important regulatory component of Zn-mediated signaling pathway. Zn activates mitogen activated protein kinases (MAPK) including ERK1/2, JNK and p38 [114,115,116], as well as tyrosine kinases, Src/EGFR, IRS1/2 and IGF1 receptor regulated signaling pathways [117,118,119]. These cascades are inactivated by Zn-dependent dephosphorylation [115,120]. In addition, Zn increases the affinity of both IGF-1 and IGF-2 for the type 1 IGF receptor tyrosine kinases (RTKs) [121] and Zn deficiency diminishes levels of insulin-like growth factor-1 (IGF-1) [122]. Zn chelation with the cell impermeable Zn chelator, DTPA (diethylenetriaminepentaacetate), partially abolishes IGF-1 stimulation of MAPK [123]. On the other hand, Zn stimulates protein tyrosine phosphorylation and MAPK activity [114] and epidermal growth factor (EGF) receptor phosphorylation and MAPK activation [119]. Zn stimulation of tyrosine phosphorylation is partially due to the inhibition of various protein tyrosine phosphatases [117], which are inhibited by Zn [118] with constants as low as 15 nM [117,124].

Apoptosis: Zn deficiency also manifests as disrupted growth factor-mediated signaling and apoptosis [125]. Under normal conditions, signaling mediated via RTKs phosphorylate and activate pro-survival and mitogenic kinases such as AKT and ERK serine-threonine kinases [29]. In turn phospho-AKT and ERK modulate downstream pathways of cell proliferation, survival and death by phosphorylating the pro-apoptotic protein BAD and facilitating its sequestration in the cytosol by the “14-3-3 proteins” [126]. The 14-3-3 proteins are regulatory proteins that bind to phosphorylated serine/threonine motifs and control multiple cellular functions including cell cycle, apoptosis, signal transduction, malignant transformation, cellular metabolism, vesicular transport, DNA replication and repair [127]. Also, the pro-survival proteins, BCL-2 and BCL-X_L_, and pro-apoptotic BCL-2 family of proteins, BAX and BAD [125] are central to activation of mitochondrial outer membrane permeabilization and resultant loss of cytochrome *c*. Under normal conditions, BAX and BAD are localized in the cytosol, while BCL-2 family members are located in the outer mitochondrial membrane [125]. Apoptotic stimuli trigger BAD dephosphorylation thereby facilitating its release from 14-3-3 proteins, regulating the conformational activation of BAX, promoting the migration of both BAD and BAX to the mitochondria [125]. BAD/BCL-2 heterodimerization sequesters BCL-2 and results in BAX permeation of both the outer and inner mitochondrial membranes resulting in the loss of the inner mitochondrial transmembrane potential (ΔΨ_m_) and release of cytochrome *c*, and downstream activation of the caspase cascade culminating in caspase-3 cleavage [125]. In parallel, Zn deficiency increases maintenance of the hypo-phosphorylated forms of AKT and ERK allowing for BAD translocation to the mitochondria, and BAX and BAD/BAX mediated cell death via activation of the intrinsic cell death pathway [128]. Further, Zn deficient cellular conditions may act to directly activate caspases [125]. Zn deficiency-induced decline of the inner mitochondrial transmembrane potential is followed by caspase-3 activation [129], which suggests that Zn regulates caspase activity [130,131,132]. TPEN-mediated Zn chelation increases cytosolic caspase-3 activity [133]. In addition, treatment of the human breast cancer cell lines MCF-7 and MDA-MB-468 with both TPEN and DTPA, activates caspase-9 and the intrinsic apoptosis pathway [134].

Conditions of Zn deficiency promote an environment conducive for the production of ROS which could also contribute to apoptosis induction [125]. Zn deficiency-induced oxidative stress occurs in association with iron accumulation in protein sites previously occupied by Zn. This can induce reactions that lead to the formation of ROS which damages cellular macromolecules [135]. Mitochondria also produce ROS, leakage of which can occur as a consequence of chemical and physical damage, or via breech of pore forming proteins such as BAX [136]. In contrast, breast cancer cells are unique in that mechanisms that regulate the ability of malignant breast cells to accumulate high levels of Zn also confer protection from undergoing apoptosis [52,137]. Zn-induced apoptosis in MCF-7 cells required the presence of functional p53 expression and p53 translocation to mitochondria, dissipation of the mitochondrial membrane potential and mitochondrial translocation of Bax [138]. Additionally, Zn-induced apoptosis in MCF-7 cells was shown to be due to a p53/ROS dependent function since p53 negative breast cancer cell lines did not undergo apoptosis in response to Zn [138]. Conventional breast cancer therapeutics have already begun, perhaps unknowingly, to target Zn regulated pathways of cell death. Tamoxifen activates ROS-mediated oxidative stress, promotes Zn accumulation in acidic autophagic vacuoles, and potentiates lysosomal membrane permeabilization and cathepsin D release [139].

## 7. Zinc Regulation of MMPs in Mammary Gland Development and Cancer

MMPs play a central role in normal physiological conditions, such as proliferation, cell motility, remodeling, wound healing [140,141]. MMP overexpression and activation contributes to tumorigenesis via promoting various pathological conditions such tumor invasion, metastasis and angiogenesis [140,141]. MMPs are a subfamily of Zn-dependent matrix metalloproteinases characterized by their HEXXHXXGXXH Zn-binding motif [140]. MMPs mediate proteolysis of extracellular matrix (ECM) components and numerous other proteins, which facilitates movement of cells through ECM, and cleaves cell-ECM adhesion proteins and cell-cell junction proteins. MMP activation promotes cell migration and invasion of breast cancer cells [142]. In humans there are 26 different MMP proteins which are all characterized by their multi-domain structures [143]. Most MMPs are secreted as latent precursors, which are proteolytically activated in the pericellular and extracellular space [140]. The MMP propeptide domain contains an unpaired cysteine residue, which folds over and interacts with the Zn ion present in the catalytic domain to maintain the enzyme in its latent form (also referred to as the “cysteine-switch”) [141]. Disruption of the cysteine-Zn interaction causes protein unfolding which activates the enzyme exposing the active site to the ligand [141]. Once mature, extracellular MMP activity can be inhibited via binding of TIMPs, tissue inhibitors of MMPs [141]. TIMPs non-covalently bind MMPs associated with the catalytic site of the mature enzyme [144]. Strictly conserved cysteine residues in the TIMP protein are necessary for chelating Zn ion in the active site of MMP thereby controlling its activity [145]. Structural changes during ductal development in the normal mammary gland as well as remodeling during pregnancy, lactation and involution involves MMP-mediated breakdown and re-synthesis of ECM components, whereas pathological changes such as mammary tumor growth and invasion also involves ECM disruption [140]. The cysteine switch may also be disrupted by oxidation of cysteine by reactive oxygen species, thereby disrupting the thiol-Zn interaction, which leads to allosteric relocation of the pro-domain, leading to activation of the enzyme with the pro-peptide still attached [146].

Deregulation of MMP functions in cancer plays a significant role in tumor invasion and metastasis, activities which are highly dependent on Zn binding to the catalytic site [146]. Therefore, here we briefly touch on early steps of MMP synthesis and acquisition of Zn as a catalytic factor. During biosynthesis, numerous Zn requiring enzymes, including MMPs, co-localize with Zn pools in the early secretory pathway [147]. It is likely that Zn transported into these compartments is used for metallation of the catalytic domain of the newly synthesized MMPs before being secreted, although direct evidence is lacking [147]. From the viewpoint of increased cellular Zn concentrations in breast cancer cells, whether changes in Zn concentrations in the secretory compartment affect MMP activation have not been addressed, and whether these changes affect the affinity of MMP for Zn is currently unknown.

## 8. Regulation of Cellular Zinc Homeostasis

Zn is an abundant and essential trace element found in relatively high concentrations in all body tissues and secretions. Total body Zn content is about 1.4–2.3 g [26]. Zn is compartmentalized within intracellular organelles including the nucleus, endoplasmic reticulum, Golgi apparatus, endosomes/lysosomes and the mitochondria [148,149,150]. Many cell types also contain vesicular structures called zincosomes that sequester high amounts of Zn and release it upon stimulation by multiple signals including growth factors [52,117]. It is important to note that “free Zn” is negligible as Zn is most often found associated with proteins and small molecular weight ligands. Despite these details, the function of Zn in subcellular compartments is still not clearly defined [151]. Zn-specific fluorophores suggest that labile nuclear Zn is significantly below cytoplasmic levels [152,153]. The development of ratiometric Zn-selective sensors for quantitatively measuring intracellular ions are beginning to improve our understanding [154]. Using a mitochondria-specific ratiometric probe, mitochondrial Zn concentration is calculated to be ~70 pM [151]. Fluorescence resonance energy transfer (FRET)-based genetically encoded metal-ion biosensors are also emerging as important tools to visualize metal-ion dynamics in live cells, including those of Zn-based sensors [155]. Multidimensional visualization of target molecules with the use of these biosensors is rendered complex considering their dynamic range and response kinetics, both properties which affect their spatial as well as temporal resolution. Use of these tools was used to specifically resolve cellular heterogeneity of Zn pools as multiple subpopulations, which could be further subdivided into populations exhibiting dynamic FRET response (expected Zn response), as well as subpopulations exhibiting static FRET response (negligible or weak Zn response) [155]. It has been suggested that cellular energy expenditure to minimize nuclear labile Zn on a level below that of cytoplasmic concentrations, and further regulate cytoplasmic vesicular sequestration, plays a role in keeping check on the effect of labile Zn in multiple cell functions, signaling pathways and apoptosis induction [156]. Insight into the regulation of Zn distribution on cell function is critical to improving our understanding of the role of Zn dysregulation in disease.

Intracellular Zn compartmentalization is tightly regulated by two families of Zn transporters [148,157]. The ZnT (SLC30A) family (ZnT1-ZnT10) of Zn transporters regulates intracellular Zn levels by transporting Zn from the cytoplasm into the lumen of organelles or across the cell membrane into the intracellular milieu [148,157]. In contrast, the ZIP (SLC39A) family (ZIP1-ZIP14) of Zn transporters, is responsible for increasing cytoplasmic Zn levels by transporting Zn into the cytoplasm from within the lumen of organelles or across the cell membrane [148,157]. In this review, we discuss intracellular Zn regulation, functional expression and localization of the two families of Zn transporters with respect to what has been reported about their dysregulation in breast cancer.

## 9. Zinc Transporters Associated with Breast Tissue Carcinogenesis

Normal mammary gland development and function is highly dependent on Zn homeostasis [148] which is necessary for the tight coupling of cell proliferation [158] and programmed cell death [125]. The Zn transporting network in the mammary gland is unique in that it plays a dual role in maintaining normal Zn levels for basic cellular Zn requirements coupled to Zn secretion during lactation and is under the control of the lactogenic hormones such as prolactin [148]. Since the signaling pathways that modulate cell growth in cancer cells also abrogate the controls for cell death, understanding Zn dysregulation in the context of breast cancer development could be important in prevention, diagnosis, targeted therapeutics design and management of the disease [148]. Examination of breast cancer biopsies as well as cultured cells have shown abnormal expression of multiple proteins that play a role in Zn homeostasis, including ZIP6 [51], ZIP7 [52], ZIP10 [50], and ZnT2 [159]. Here we discuss what is currently known about the functional defects in these individual components of the Zn regulatory network and downstream consequences of which could promote breast cancer development. In addition, we compare deregulation of specific Zn transporters in breast cancer with other hormonally regulated cancers, such as prostate, pancreatic and ovarian cancers, as reported in current literature. 

ZIP6: Normally, ZIP6 localizes to the plasma membrane of mammary epithelial cells and imports Zn into the cytoplasm [148,157]. Upregulated ZIP6 protein levels in tissue biopsies from breast cancer patients are positively correlated with estrogen receptor (ER+) [160,161], and is also a marker for the luminal A subtype of clinical beast cancer [162]. Additionally, ZIP6 protein expression is upregulated in estrogen treated MCF-7 and ZR-75 breast cells [163,164], suggesting ER modulated dysregulation of Zn homeostasis [148]. High levels of ZIP6 expression is found in metastatic breast cancer cells [165,166] and is positively correlated with lymph node metastasis [167,168], suggesting the possibility that ZIP6 plays a role in progression.

Mechanisms through which ZIP6 restricts metastasis are thought to be related to its ability to abrogate cell to cell contacts via modulation of epithelial-mesenchymal transition (EMT) [169]. During zebrafish embryonic development specifically the gastrula stage, ZIP6 expression is dependent on the STAT3 transcription factor [170]. Further, ZIP6 regulates the nuclear translocation of the Zn-finger protein Snail, which is a transcriptional repressor of the epithelial adhesion molecule E-cadherin [171,172]. ZIP6 over-expression in breast tumors and cancer cell lines is correlated with phosphorylated (activated) STAT3 [173] and Snail expression in breast tumor biopsies is positively associated poor survival [174,175,176]. ZIP6 controls EMT by decreasing E-cadherin in the MCF7 [177] and T47D [178] cells. Importantly, high ZIP6 protein levels correlate with less aggressive tumors [179] thus ZIP6 has been suggested to play a role in potentially modulating constraining mechanisms relating to tumor proliferation and adhesion [178].

Lastly, a unique characteristic of ZIP6 is the highly conserved putative metalloprotease motif (HEXPHEXGD) that resembles the active site motif found in MMPs [166,180,181]. Increased expression levels of certain MMPs are associated with tumor growth, invasion, metastasis and angiogenesis and correlates with poor prognosis [182]. Expression of MMPs is also upregulated during EMT and correlates with tumor cell invasion and metastatic potential [183]. MMPs can disrupt cell adhesion by processing components of cell-cell and cell-extracellular matrix contacts, and interference with functions of E-cadherin, which are specifically associated with epithelial cell-cell adhesion [183,184,185]. MMP processing of E-cadherin and its downregulation are critical steps in the initiation of the EMT process [186] and contributes to migration and metastases of cancer cells [183,187,188]. Further investigation is needed to examine the mechanistic role of ZIP6 in the regulation of cellular Zn pools, cellular adhesion and their inter-relationship in breast cancer transition and/or metastasis.

ZIP7: In normal cells, the intracellular free Zn concentration changes in response to extracellular stimuli resulting in cellular changes that are categorized as “early” and “late” events [189]. Early Zn signaling is shown to originate from the endoplasmic reticulum and is dependent on calcium influx and MAPK activation, and was observed several minutes after stimulation [24]. Thus released, free Zn levels could activate/suppress intracellular signaling molecules such as tyrosine phosphatases [117]. In contrast, late Zn signaling is detected several hours after extracellular stimulation and is dependent on transcriptional regulation of Zn transporter expression [24]. Intriguing evidence suggest that the Zn transported by ZIP7 acts like an early wave intracellular second messenger [24]. ZIP7-mediated Zn released from the endoplasmic reticulum activates tyrosine kinase [52]. Tyrosine kinase activation results from Zn-dependent association of ZIP7 with the protein kinase CK2, with simultaneous CK2 mediated phosphorylation of ZIP7 on Ser^275^ and Ser^276^ residues [190]. Since targeting ZIP7-mediated Zn release in breast cancer cells led to loss of cell proliferation control and invasion [52], CK2 regulated phosphorylation of ZIP7 suggests the possibility of targeting CK2 for treatment of breast cancer [190].

ZIP7 expression has been associated with breast cancer progression [52]. Studies using the MCF-7 and tamoxifen-resistant (TamR) human breast cancer cell lines showed that ZIP7 mediates a greater increase in intracellular Zn levels in the TamR cell line compared with the non-TamR counterparts, and consequently leads to activation of EGFR, Src, and IGF-1R regulated signaling pathways, promoting growth and invasion [52,162]. Under these conditions, both ZIP7 and EGFR are overexpressed and downregulation of ZIP7 decreases intracellular Zn levels and reduces cell migration through inactivation of EGFR-mediated signaling [162]. In a related line of reasoning, EGFR activation correlates with neoplastic progression and its over-expression in tumors has been correlated with poor survival [191]. It is of note that since ZIP7 localizes to the endoplasmic reticulum/Golgi membrane, ZIP7-mediated Zn movement from these compartments may activate tyrosine kinase regulated signaling pathways [192]. 

ZIP10: Like ZIP6, ZIP10 expression is related to metastatic breast cancer [157]. Tumor samples derived from breast cancer biopsies show a significant association between ZIP10 mRNA expression with metastasis to the lymph node [50]. Increased ZIP10 mRNA levels are expressed in highly invasive breast cancer cell lines such as MDA-MB-231 and MDA-MB-435S [50]. Further, attenuation of ZIP10 expression and treatment with the Zn-chelator TPEN correlates with decreased intracellular Zn and migration in MDA-MB-231 cells [50]. These studies support the role of ZIP10 for migration potential in breast cancer cells. Greater understanding of ZIP10/Zn associated mechanisms related to cell signaling and motility remains to be elucidated. 

ZnT2: ZnT2 is abundantly expressed in the mammary gland and is over-expressed in ER + T47D cells [159,193,194]. T47D cells are essentially devoid of metallothionein expression and as a result cannot buffer free Zn in the cytoplasm. Hyperaccumulation of Zn in the malignant T47D breast tumor cells is correlated with ZnT2 overexpression and increased vesicular Zn pools [159]. Further, attenuation of ZnT2 increases cytosolic Zn pools and induces autophagy, suggesting that abundant ZnT2 expression in malignant cells protects the metallothionein-null breast tumor cells from Zn-induced cytotoxicity by redirecting Zn into vesicular compartments [159]. Since malignant breast cancer cells accumulate Zn [45,46], and exposure to high levels of Zn activates apoptosis [195], mechanisms have evolved to protect cells against Zn modulated cell death. Two genetic variants of ZnT2 have been characterized that were subsequently shown to dysregulate Zn transport in normal mammary epithelial cells, which may further implicate ZnT2 dysfunction with breast disease [149].

## 10. Dysregulation of Zinc Metabolism in Other Hormonally Regulated Cancers

In recent years, increasing evidence has implicated abnormal Zn homeostasis in the development of multiple cancers, including prostate, pancreatic and ovarian cancers. However, no common mechanisms have been established with respect to Zn dysregulation and cancer development. Interestingly, a diverse group of ZIPs other than ones that are associated with breast cancer, have also been reported to play a role in regulating cell proliferation and apoptosis in prostate, pancreatic and ovarian cancers. This suggests that Zn dysregulation in cancer is cell type specific. Although the different cancers showed differential sensitivity to Zn exposure and ability to undergo cell death, a common target appears to be the activation of the intrinsic apoptotic pathway by Zn exposure at the level of the mitochondria. In order to highlight similarities and differences observed in Zn transporter expression and function in breast cancer compared with other hormonally regulated cancers, we present a brief discussion on what is known about deregulation of other Zn transporters and the relationships to defects in Zn metabolism in prostate, pancreatic and ovarian cancer. Overall, these studies highlight the observation that cells derived from different cancer types appear to display varying thresholds for Zn exposure and Zn regulated apoptosis induction.

Prostate cancer: Zn dyshomeostasis in prostate cancer is opposite in nature to that observed in breast cancer. Prostate tissue contains the highest Zn concentration of all soft tissues [196]. Normal human prostate glandular epithelial cells accumulate high levels of Zn via ZIP1 regulated uptake [197,198], in addition to activities of ZIP2 and ZIP3 [199]. Normally, Zn accumulation in prostate cells inhibits growth and proliferation, as well as invasiveness/motility and induction of mitochondrial apoptosis [199]. ZIP1 is localized to the basal membrane and appears to be important for Zn uptake from the circulation as the primary source of cellular Zn, whereas ZIP2 and ZIP3 are localized predominantly to the apical membrane [199]. Although abundant ZIP1, ZIP2 and ZIP3 expression is also observed in hyperplastic prostate epithelial cells, their respective protein levels are downregulated in malignant cells [200]. Cellular Zn levels are significantly decreased in prostate cancer [196] and downregulation of transporter expression in malignant tissue correlates with marked depletion of Zn levels [200]. Since high Zn levels impose tumor suppressive effects in normal prostate cells, gene silencing of these transporters is required for malignant progression. Since downregulation of all three transporters is associated with loss of Zn and malignancy [199], ZIP1, ZIP2 and ZIP3 are regarded as tumor suppressors in prostate cancer [201]. In addition, extracellular Zn is thought to play a role in cell signaling. Recent studies have reported that the extracellular Zn concentration can be sensed by cell membrane G-protein-coupled receptors(s) called ZnR [202]. More recent studies suggest that an orphan G-protein-coupled receptor GPR39 is a Zn receptor which is differentially and spatially expressed in specific regions of the mouse prostate, suggesting that GPR39 could play an important role in sensing Zn for normal prostate functions [203]. In addition, recent studies directly link the role of GPR39 in mediating Zn signaling via activation of the MAPK pathway leading to downstream activation of epithelial repair [204].

In normal prostate cells, the anti-tumor mechanisms resulting from high intracellular Zn levels are a consequence of a regulatory step in intermediary mitochondrial metabolism and bioenergetics [205,206]. High levels of mitochondrial Zn accumulation is necessary for non-malignant prostate cells to synthesize, accumulate and secrete high levels of citrate into the prostatic fluid, a major function of the prostate gland [206]. In the mitochondria, Zn inhibits the activity of the enzyme m-aconitase, which prevents citrate oxidation via the Krebs cycle [205,207,208,209]. This is in contrast to most other mammalian cells where citrate oxidation is an essential bioenergetic step and complete glucose oxidation yields 38 ATP molecules [205,207,208,209]. Malignant prostate cells which have lost the ability to accumulate Zn undergo a metabolic transformation from citrate-producing to citrate-oxidizing cells [205,207,208,209]. In malignant cells, the absence of high mitochondrial Zn levels lowers the inhibitory threshold which activates m-aconitase, leading to complete oxidation of glucose [205,207,208,209]. Additionally, in normal cells, Zn inhibits terminal oxidation in mitochondria at the level of Complex I and/or II [210]. In contrast, constitutive activity of complexes I-IV in malignant prostate mitochondria are 50%*–*80% lower than in liver mitochondria [210,211]. Thus, the process of high levels of Zn accumulation and its inhibitory effects on citrate oxidation are unique metabolic activities in the prostate gland and relate to Zn regulation of tumor suppression activities. Prostate mitochondria sequester as much as 30% of cellular Zn [212], however, mechanisms of Zn uptake/transport into these mitochondria are as yet unknown. Low molecular weight Zn ligands (<10 kDa) such as metallothionein, citrate, aspartate and histidine constitute the source of Zn-binding and transport into the mitochondria [56,212,213,214,215,216]. In the case of prostate cells, citrate is a ligand for Zn [217]. A putative mitochondrial Zn uptake transporter activity capable of importing Zn from cytosolic Zn ligands has been identified in prostate mitochondria but remains to be characterized [210,216]. Thus, in addition to downregulated expression of ZIP1, ZIP2 and ZIP3, the decline in mitochondrial Zn levels in malignant prostate cells is also attributed to reduced citrate levels which function as the major Zn ligands. 

Prostate cancer cells exposed to Zn undergo apoptosis and is a direct effect of Zn on mitochondria resulting in cytochrome *c* release and caspase-3 activation [218,219]. Studies show that this Zn mediated effect on mitochondria is due to increased Bax-mitochondrial interaction and Bax-induced pore formation which preceded cytochrome *c* release [220]. Zn differentially increases the levels of Bax without increasing levels of Bcl_2_, increasing the Bax/Bcl_2_ ratio, indicating death signals which favor apoptosis induction [220]. The anti-tumor effects of high Zn levels inhibit invasiveness and metastasis of malignant prostate cells [221]. Aminopeptidase N purified from human prostate was irreversibly inhibited by Zn suggesting that this protein could be associated with invasive capacity [222].

Ovarian cancer: Abnormal Zn homeostasis in ovarian cancer cells display similarities to malignant prostate cancer cells. Intracellular Zn levels in ovarian tumor tissues are also significantly lower than the levels found in benign tissues [223]. It is also noteworthy that patients with ovarian cancer exhibit lower serum Zn levels compared with normal controls [33], similar to patients with breast cancer [37,38]. There is very limited information regarding the relationship between Zn dyshomeostasis and ovarian cancer. Expression and functions of Zn transporters in relation to decreased cellular Zn levels and related mechanisms to progression of ovarian cancer have yet to be explored. Similar to prostate cancer cells, Zn treatment of the OVCAR-3 ovarian cancer cells increases Zn accumulation and activates apoptosis, which is regulated through increased Bax/Bcl_2_ ratio and activation of caspase-3 [224]. More specifically, treatment of A2780 ovarian cancer cell line with Zn ionophores induces apoptosis and necrosis, and is associated with Akt and NF-kappaB signaling pathways [225]. As both proteins normally regulate pathways promoting cell survival, this provides evidence that cellular Zn levels play a critical role in ovarian cancer progression [225].

Pancreatic cancer: A recent study addressed the role of Zn and ZIP4 in the development of pancreatic cancer [57]. ZIP4 expression is markedly increased in malignant tissue compared to the surrounding normal tissue and is expressed in pancreatic cancer cell lines. In vitro studies show that ZIP4 expression is correlated with increased cellular Zn accumulation and increased cell proliferation [57], suggesting similarities to abnormal Zn homeostasis in breast cancer cells. The same study also showed increased ZIP4 expression in xenografts that correlates with increased tumor Zn levels and tumor growth [57]. The association of ZIP4 in the development and progression of pancreatic cancer is a surprising observation, since ZIP4 is normally expressed along the entire gastrointestinal tract and functions in the uptake of dietary Zn from the apical membrane [226]. The potential role of ZIP4 as an anti-apoptotic protein was shown using RNAi knockdown in mouse Hepa cells, which results in increased apoptosis and decreased migration, whereas its overexpression in Hepa and MCF-7 cells enhances migration [227]. Clinical evidence suggests that liver tissues derived from patients with hepatocellular cancer contain significantly lower levels of Zn than the corresponding normal tissues [201,228], suggesting similarities with malignant prostate cells. A more recent study reported that silencing of ZIP3 gene expression was associated with pancreatic adenocarcinoma [229]. Loss of ZIP3 was correlated with loss of Zn in ductal and acinar epithelium in adenocarcinoma compared with normal epithelium [229].

The effect of Zn treatment on pancreatic cancer cells has shown conflicting results. In one study, Zn inhibited caspase-3 and caspase-8 activation and decreased ratios of pro-apoptotic *versus* anti-apoptotic proteins of the Bcl_2_ family displaying growth-promoting/anti-apoptotic effects [230]. Treatment of these cells with TPEN induced Zn depletion and apoptosis induction. Another study showed that Zn treatment of pancreatic cancer cells induced cellular Zn accumulation, increased ROS production and apoptosis induction [231]. In this study, Zn regulated ROS production was shown to cause nuclear translocation of AIF and resulted in apoptosis induction, thereby completely bypassing mitochondrial activation of caspase-3 and caspase-8 activation. The disparity in the outcome of the two studies is unknown.

## 11. Perspective

Normal pathways of Zn regulation play an important role in a multitude of cellular housekeeping functions. Thus, the ubiquitous nature of Zn creates a difficult task when attempting to isolate specific pathways that are deregulated in breast cancer. The ability of Zn to regulate oxidative stress in cells has the potential to modulate functions of redox-sensitive proteins, as well as induce oxidative modifications on damaged DNA. Zn regulation of DNA damage response/repair pathways and their regulatory link with Zn-dependent cell proliferation/cell death pathways deserves a fresh approach with respect to development of targeted therapeutics. On the other hand, deregulated Zn transporter functions have gained a lot of attention in terms of their expression and activities in modulating intracellular Zn levels and their correlation with not only breast cancer, but also other cancers in tissues that are also hormonally regulated. Control of intracellular Zn fluxes is a dynamic process, and the role of Zn transporters in regulating availability of Zn to regions of specific cellular functions suggest that subcellular compartmentalization of Zn transporters in vicinity of their target proteins/signaling pathways could play a critical role in timing of Zn-dependent mechanisms. Differences in the regulation of such compartmentalization could also drive cell-specific differences. Abnormalities in cellular sorting mechanisms that determine compartmentalization of Zn regulated functions could potentially result in tumorigenesis. In this respect, abnormal functioning of both ZnTs as well as ZIPs and imbalances in their activities could play a role in Zn dyshomeostasis and cancer development. This may provide the potential for identifying novel targets for not only breast cancer, but other cancers that display abnormal Zn homeostasis, and should be addressed in future studies.
